# Perceptions about the causes and treatment of cancer – A cross-sectional survey of university students in Ghana

**DOI:** 10.1016/j.pmedr.2023.102160

**Published:** 2023-02-23

**Authors:** Martin Gameli Akakpo, Evelyn Owusu Roberts, Rachel Annobil, Abigail Serwaah Aboagye

**Affiliations:** Department of Human Development and Psychology, Faculty of Arts and Sciences, Regent University College of Science and Technology, P.O. Box DS 1636, Accra, Ghana

**Keywords:** Cancer awareness, Stigma, Cancer treatment, Causes, Health psychology, Cancer control, CAM, Cancer Awareness Measure, CAM-MYCS, Cancer Awareness Measure-Mythical Scale, CASS, Cancer Stigma Scale

## Abstract

Cancer awareness campaigns in Ghana and many parts of the world have gathered pace in recent years. Despite this encouraging trend, there has been no significant reduction in stigma in Ghana. This study investigated the role of beliefs about the causes of cancer in stigmatization and perceptions about treatment. Using standardized scales deployed in a survey, the study measured student perceptions about the causes of cancer, stigmatization, and opinion about the possibility of treating cancer. 225 students were sampled from two universities in the Ghanaian capital of Accra. The study used multiple linear regression and a logistic regression to answer two research questions. These asked whether beliefs in the mythical causes of cancer are associated with stigma towards cancer and whether this stigma is associated with the belief that cancer cannot be treated. The findings point to the association between perceived causes of cancer and stigma. Stigmatization was associated with the belief that cancer could not be treated. The findings imply that the perceived causes of cancer are associated with stigma and should thus be addressed by campaigners. Educating the public about the causes of cancer and addressing some of the myths can help reduce stigma and correct misperceptions about treatment.

## Highlights

• Cancer Stigmatization is associated with beliefs not regularly addressed in cancer awareness campaigns.
• Cancer Stigmatization is associated with beliefs that modern technology (such as TV/radio and microwaves) causes cancer.
• Stigmatization is associated with the belief that cancer cannot be treated.
• Stigmatization is a barrier to cancer awareness campaigns amongst higher education students.

## 1 Introduction

### 1.1 Background

Public perceptions about the causes of cancer play an important role in behavior toward treatment (Shahab et al., 2018, Osei-Afriyie et al., 2021). Negative perceptions and blaming of patients are related to stigmatization/stigma towards patients and disbelief in treatment (Else-Quest & Jackson, 2021; Soffer, 2022; Larkin et al., 2021). Despite the numerous awareness campaigns and entire months of dedicated publicity, cancer continues to be a burden on Ghana’s health system (Osei-Afriyie et al., 2021). Detection is often late, leading to a high rate of deaths in comparison to developed countries. Many studies have identified stigmatization as a barrier to treatment-seeking behavior and advise more research to understand and address stigmatization.

One such study was by Maree and Wright (2011) who conducted a survey of 105 participants in poorer suburbs in Tshwane, South Africa. Stigmatization linked to perceptions about causes was identified as a barrier to cancer treatment-seeking behavior. This finding is still relevant, 12 years on. Consistent with the findings of Maree and Wright (2011), Sheperd and Gerend (2014) used an experimental design with four conditions of 487 participants and found that blaming victims contributes to stigma. Shepherd and Gerend (2014), noticed that people who have perceptions that cancer is caused by specific human factors are more likely to stigmatize cancer patients. As recently as in 2019, Sayed (2019) deployed a mixed-method cross-sectional study of 679 adults in Kenya and found that breast cancer knowledge was related to treatment-seeking behavior. The study found that more literate participants with a better understanding of the causes of breast cancer were more likely to support screening and treatment. These and other studies such as Osei-Afriyie et al. (2021) in Ghana, Olodade et al. (2019) in Nigeria, and Oystacher et al. (2018) in South Africa recommend more studies on the relationship between perceived causes of cancer, stigmatization, and treatment-seeking behavior. Further, studies, such as a review by Larkin et al., 2022, Shahab et al., 2018 recommend more studies to investigate cancer stigmatization and the beliefs about treatment. The current study involved students with more exposure to awareness campaigns and information.

Studies investigating perceptions about the basis of cancer have used factors traditionally supported by researchers and recent causes gaining some audience within the scientific community (Bandara & Carpenter, 2020). The traditionally accepted causes include causal beliefs such as alcohol use, active and passive smoking, sunburn and having a relative with cancer (Stubbings, et al., 2009). The recently proposed causes on the other hand can be identified as mythical (Smith et al., 2018), though Bandara and Carpenter (2020) indicate that those causes have scientific support. These mythical beliefs include the use of mobile phones, cleaning products, microwave ovens, the consumption of genetically modified foods, and stress. These have received some audience with the increased exposure to electromagnetic frequencies, air pollution, and food preservatives amongst other modern occurrences (Bérard et al., 2015). In this study, we used both the traditionally accepted causes and the ‘mythical’ causes to find their relationship with stigma. This is supported by previous studies such as the work of Munir et al. (2022) which used both the traditionally accepted causes and ‘mythical’ causes of cancer. In their study of 657 individuals in Pakistan, it was observed that the mythical causes were less known to participants and thus influenced stigmatization. This was like the findings of Paytubi et al. (2022), who in their cross-sectional survey of 1494 respondents in the United States, found a strong link between mythical causes and resistance to cancer prevention or treatment measures.

Cancer stigma is common and usually takes the form of disbelief in treatment, self-blame, and negative behavior toward cancer patients (Else-Quest and Jackson, 2014, Lee and Shi, 2022). In some health systems, cancer patients are stigmatized, and some individuals equate cancer to death (Fujisawa and Hagiwara, 2015, Oystacher et al., 2018). This results in difficulties for cancer awareness campaigns (Osei-Afriyie et al., 2021). Despite the abundance of online information and ‘Dr. Google’s resources (Akakpo, 2022), awareness has remained low (Shahab et al., 2018). Stigma toward cancer can be exacerbated by beliefs about the causes and culture among others (Ololade et al., 2019, Noronha, 2020). The mass media is a key source of information (Akakpo, 2022) that shapes stigma (Soffer, 2022). A key component of cancer stigma is patient-blaming and the notion that the outcome of a positive diagnosis is always disastrous (Marlow & Wardle, 2014). Using suggestions from previous studies, this study formulated objectives which are listed in the next section.

### 1.2 Objectives

• Investigate participants’ perceptions about the causes of cancer.
• Explore the relationship between the type of perceived causes of cancer and stigmatization.
• Determine the relationship between stigmatization and the belief that cancer is treatable.

## 2 Methods

### 2.1 Study design

A cross-sectional survey was used to collect data in the second week of March 2022. Researchers approached students after lectures and explained the purpose of the research. Afterward, students who decided to participate remained in the lecture hall. Participants had the option of filling out the paper or online questionnaires. Students who opted to respond on their smartphones were sent a link to the questionnaire which was hosted on the NubiaMetrics online survey tool. Participants were given 5 – 10 minutes to complete the questionnaires.

### 2.2 Setting

Questionnaires for the study were distributed at the lecture halls of two universities in Accra, Ghana. These universities are the University of Ghana, Legon, and the Regent University College of Science and Technology. These universities are amongst the most frequent beneficiaries of cancer awareness campaigns.

### 2.3 Participants

The sample was drawn from university students enrolled in the selected universities in Accra. In Ghana, Cancer Awareness campaigns by health experts are more common in cities than in rural areas with university students in Accra being frequent beneficiaries. This makes students in Accra an ideal group for this study investigating perceptions about cancer. A total of 225 individuals responded to the survey after 400 were approached. To participate in the study, the individual was required to be enrolled in any non-medical undergraduate degree program. To deal with bias, the study excluded students of medicine, nursing, biomedical science, and related fields. The study, therefore, sampled students who only know about cancer from personal experience or public awareness campaigns.

### 2.4 Measurement

The questionnaire used to collect data (see Appendix A), was in English which is the language of instruction for undergraduate programs in the selected universities. The scales used to measure perceived causes of cancer were the Cancer Awareness Measure (CAM) by Stubbings et al. (2009) and the Mythical Causes Scale (CAM-MYCS) developed by Smith et al. (2018). Stigma towards cancer was measured with the Cancer Stigma Scale (CASS) developed by Marlow and Wardle (2014). All measures were administered on a 5-point Likert scale ranging from *Strongly Disagree* which scored ‘1′ to *Strongly Agree* which scored ‘5′. The possible range of scores for the 11-item CAM is 11 to 55 and in this study, the scale yielded a Cronbach alpha reliability coefficient of 0.63. The Cronbach alpha reliability for the CAM-MYCS was 0.83. This was a 12-item scale with a possible range of scores from 12 to 60. The possible range of scores on the 9-item CASS was 9 to 45, with a Cronbach alpha reliability of 0.75 in this study. Non-scale measures used were age, gender, study program, family history of cancer, belief in cancer treatment, and interest.

### 2.5 Study size

Based on an estimated population of 200,000 undergraduate students in the city of Accra, the required sample size (out of 200,000) at a 90% confidence level and 5% margin of error was 274. The number of undergraduate students in Accra was advised by an aim to sample individuals with a perceived high literacy and more exposure to awareness information. After approaching 400 students, 225 complete responses (56.25%) were obtained. Researchers were unable to increase the sample size due to the limited availability of students within the research period and limited resources.

### 2.6 Statistical methods

To understand the range of scores on variables, univariate analysis was conducted. This resulted in descriptive statistics such as Cronbach alpha reliability, minimum to maximum scores, means, and standard deviations (see Table 1). A Pearson product-moment correlation coefficient was computed to explore the relationship between scores on stigmatization and perceived causes of cancer (see Table 1).

The first research question which aimed to investigate whether *beliefs in the mythical causes of cancer are associated with stigmatization* was tested with multiple linear regression. The independent variable in the model was beliefs in the mythical causes of cancer (CAM-MYCS) and the causes of cancer (CAM) while stigmatization (CASS) was the dependent variable. The second research question which sought to find out whether *stigma towards cancer is associated with perceptions about the treatment of cancer* was tested with logistic regression. In this model, stigmatization was the independent variable while perceptions about the treatment of cancer was the dependent variable. All data were analyzed with SPSS version 26. All 10 incomplete responses were excluded from the final data analysis and the final count of 225. Responses were deemed incomplete if the individual did not entirely complete the CAM, CAM-MYCS, and CASS.

### 2.7 Ethical considerations

The introductory page of the questionnaire contained the informed consent section. Students who were not willing to participate were allowed to leave the lecture hall without any consequence. Participation in the study was voluntary and respondents were assured that the responses will be kept confidential. All data were anonymous and private information such as names, phone numbers, or email addresses were not collected. The study was granted an exemption from ethical review because it was an anonymous survey of students with no introduction of harmful or manipulated variables. Researchers kept their academic superiors informed about the nature and progress of the study.

## 3 Results

### 3.1 Participants

The 225 respondents who completed the questionnaires were aged between 18 and 54 with a mean age of 23.17 and a standard deviation of 5.50. There were 144 (64.9%) female respondents and 78 (35.1%) males, while some respondents did not complete the gender item.

### 3.2 Descriptive data

**Table 1:** Descriptive statistics, and correlations, of scores on perceived causes of cancer and stigma towards cancer.

Variables	L/H	M	SD	1.	2.	3.
1. CAM (N = 206)	17/52	31.11	5.75	–		
2. CAM-MYCS (N = 211)	12/57	37.38	7.39	0.41*	–	
3. CASS (Stigma) (N = 217)	9/43	23.75	5.95	0.09	0.23*	–

Note: 1 = CAM- Cancer Awareness Measure. 2 = CAM-MYCS – Cancer Awareness Measure – Mythical Causes Scale. 3 = Cancer Stigma Scale (CASS). N = Included cases. L/H = Lowest score/Highest score, M = Mean. SD = Standard deviation, * = significant (p) < 0.05.

### 3.3 Main results

The study answered two research questions with multiple linear and logistic regressions.

#### 3.3.1 Is the belief in the mythical causes of cancer, associated with cancer stigma? (N = 206)

A significant model emerged from a linear regression (*R = 0.22, R^2^ = 0.05, Adjusted R^2^ = 0.04, Standard Error = 5,89, R^2^ change = 0.05*). High scores on the mythical causes of cancer scale corresponded with a high score in stigma. The model was supported, and an ANOVA (*degrees of freedom = 2, 187, F = 4.66, p =.01*) indicated that differences in scores were linked to the CAM-MYCS. This effect size from the model was significant but small (*r^2^ = 0.05*) indicating that the model explains only some variance in the data. Coefficients (see Table 2) indicated the contribution of scores on CAM-MYCS to the variations in the CASS. The negative relationship between scores on the CAM and CASS was not significant.

**Table 2:** Coefficients of predictors of Stigma (CASS).

Variables	B	SE	β	Sig (p)
CAM	-0.04	0.08	-0.04	0.63
CAM-MYCS	0.19	0.07	0.23	0.00

Note: CAM = Cancer Awareness Measure. CAM-MYCS = Cancer Awareness Measure – Mythical Causes Scale, B = Unstandardized coefficient, SE = Standard Error, β = Beta, Sig (p) = Significance (p-value).

#### 3.3.2 Is stigma towards cancer associated with perceptions about the treatment of cancer? (N = 213)

After testing for assumptions, a logistic regression analysis was used to answer this research question. Scores on stigma contributed to the model. The unstandardized Beta weight for the Constant was *B = 4.44, SE = 0.94, Wald = 22.10, p =.00*. The unstandardized Beta weight for the model was, *B = -0.12, SE = 0.04, Wald = 10.94, p =.00*. The estimated odds ratio (used as the effect size) favored a decrease in the perception that cancer can be treated for every increase in scores on stigma *[Exp (B) = 0.891, 95% CI (0.83, 0.95)]*.

## 4 Discussion

### 4.1 Key results

There are two main findings from this study.

Firstly, individuals who have strong beliefs in the mythical causes of cancer show stigma towards cancer. This is like previous findings of studies conducted in the United States and Pakistan by Paytubi et al., 2022, Munir et al., 2022 which suggest that beliefs in the mythical causes of cancer are associated with stigma. Similar studies in Kenya (Sayed et al., 2019), and Nigeria (Ololade et al., 2019) support this finding from Ghana as well. These causes have not been adequately addressed by researchers and awareness campaigners. In the current study, beliefs in the causes listed in the CAM did not produce high scores on stigma. An understanding of these causes allows people to accept that even after taking all precautions against cancer, there may be a natural predisposition. This risk can be mitigated by regular screening for early detection, lifestyle modification, and prompt treatment. Stigma towards cancer is a significant impediment to the behavioral change (Lee & Shi, 2022) needed to adopt healthy lifestyles and seek treatment for cancer. Efforts to promote awareness and reduce stigma can focus on promoting knowledge about both the traditionally accepted causes of cancer and the mythical causes.

Secondly, individuals who show stigma towards cancer do not believe it can be treated. This is like the findings by Maree and Wright, 2011, Shepherd and Gerend, 2014, and Sayed et al. (2019). Efforts to reduce cancer mortality and morbidity are threatened by unaddressed mythical causes and the notion that cancer is untreatable. This finding means individuals who show stigma towards cancer are not likely to support or believe in its treatment. This posture negatively affects efforts in many health systems, including Ghana’s, to increase awareness about both causes and treatment of cancer. Like other studies (Fujisawa and Hagiwara, 2015, Else-Quest and Jackson, 2014, Soffer, 2022), the belief that cancer cannot be treated is common in those who receive insufficient awareness information. Awareness information is sufficient if it addresses both common beliefs and recent beliefs identified on the CAM-MYCS. The current study provides important knowledge to help campaigners fight stigma and increase awareness by addressing both traditionally promoted causes like alcoholism, smoking, sunburning, and genetics as well as mythical causes like exposure to electromagnetic frequencies, eating genetically modified foods, and stress.

## 5 Limitations

Findings from the study can be generalized to undergraduate students in Ghana who are exposed to cancer awareness campaigns. These are students in metropolitan areas with regular cancer awareness campaigns and entire months dedicated to specific cancers. A larger and more representative sample will make findings more generalizable. Future studies can include moderators or mediators such as demographic variables and other variables such as family history of cancer and level of education. Despite the limitations, this study contributes to knowledge about why cancer awareness campaigns should include both traditionally accepted causes of cancer and recently supported causes.

## 6 Conclusion

Findings from this work have implications for cancer awareness campaigns and efforts to reduce stigmatization. Campaigns should address both traditionally accepted causes on the CAM and the causes on the CAM-MYCS which are increasingly gaining support. The causes listed in the CAM-MYCS are popular with the public but not adequately addressed by awareness campaigns. Addressing these causes can help reduce stigma and lead to behavior modification and treatment-seeking. These findings contribute to the generation of hypotheses for studies using larger populations and more representative samples.

## CRediT authorship contribution statement

**Martin Gameli Akakpo:** Conceptualization, Methodology, Validation, Formal analysis, Investigation, Data curation, Writing – original draft, Writing – review & editing, Supervision. **Evelyn Owusu Roberts:** Conceptualization, Methodology, Validation, Writing – review & editing, Supervision. **Rachel Annobil:** Conceptualization, Methodology, Validation, Writing – review & editing. **Abigail Serwaah Aboagye:** Conceptualization, Methodology, Validation, Investigation.

## Declaration of Competing Interest

The authors declare that they have no known competing financial interests or personal relationships that could have appeared to influence the work reported in this paper.

## Data Availability

Data will be made available on request.
