# Supplementary material for: Perceptions about the causes and treatment of cancer – A cross-sectional survey of university students in Ghana
Source: Prev Med Rep. 2023 Feb 23;32:102160. doi: 10.1016/j.pmedr.2023.102160 (PMC9986619; doi:10.1016/j.pmedr.2023.102160)
Supplement: Supplementary data 1 [file mmc1.docx]

**Perceived causes and prospects of treating cancer**

Dear Respondent,

We are a team of researchers from the Regent University College of Science and Technology, Ghana. This study is investigating Perceptions about the causes and treatment of cancer.

The following questionnaire is split into three sections dedicated perceived causes of cancer, attitude toward cancer diagnosis and treatment, and demographics.

Participation in this study is voluntary and there are no rewards for completing the survey. The questions are self-explanatory but if you have any questions or concerns please do not hesitate to contact us (martin.akakpo@regent.edu.gh). There are no detrimental effects to your participation in this study.

You need 3-5 minutes to complete the questionnaire. All answers will be handled confidentially, and we will not ask for any information that can link you to your answers.

Thank You

MGA, EOR, RA and ASA

**Section 1 – Perceived causes of cancer**

*Please indicate your agreement with the following perceived causes of cancer.*

|  | Strongly Disagree | Disagree | Not sure | Agree | Strongly Agree |
| --- | --- | --- | --- | --- | --- |
| 1. Smoking any cigarettes at all. | ☐ | ☐ | ☐ | ☐ | ☐ |
| 2. Exposure to another person’s cigarette smoke. | ☐ | ☐ | ☐ | ☐ | ☐ |
| 3. Drinking more than 1 unit of  alcohol a day. | ☐ | ☐ | ☐ | ☐ | ☐ |
| 4. Eating less than 5 portions of fruit and vegetables a day. | ☐ | ☐ | ☐ | ☐ | ☐ |
| 5. Eating red or processed meat once a day or more. | ☐ | ☐ | ☐ | ☐ | ☐ |
| 6. Being overweight. | ☐ | ☐ | ☐ | ☐ | ☐ |
| 7. Getting sunburnt more than once  as a child. | ☐ | ☐ | ☐ | ☐ | ☐ |
| 8. Being over 70 years old. | ☐ | ☐ | ☐ | ☐ | ☐ |
| 9. Having a close relative with cancer. | ☐ | ☐ | ☐ | ☐ | ☐ |
| 10. Infection with HPV (Human  Papillomavirus). | ☐ | ☐ | ☐ | ☐ | ☐ |
| 11. Doing less than 30 mins of  moderate physical activity 5 times a week. | ☐ | ☐ | ☐ | ☐ | ☐ |

*Please indicate whether you think any of the following cause cancer.*

|  | Strongly Disagree | Disagree | Not sure | Agree | Strongly Agree |
| --- | --- | --- | --- | --- | --- |
| 1. Exposure to electromagnetic frequencies (e.g. TV/radio and Wi-Fi). | ☐ | ☐ | ☐ | ☐ | ☐ |
| 2. Eating food containing additives. | ☐ | ☐ | ☐ | ☐ | ☐ |
| 3. Living near power lines. | ☐ | ☐ | ☐ | ☐ | ☐ |
| 4. Feeling stressed. | ☐ | ☐ | ☐ | ☐ | ☐ |
| 5. Eating food containing artificial sweeteners. | ☐ | ☐ | ☐ | ☐ | ☐ |
| 6. Using cleaning products. | ☐ | ☐ | ☐ | ☐ | ☐ |
| 7. Eating genetically modified food. | ☐ | ☐ | ☐ | ☐ | ☐ |
| 8. Using mobile phones. | ☐ | ☐ | ☐ | ☐ | ☐ |
| 9. Using aerosol containers. | ☐ | ☐ | ☐ | ☐ | ☐ |
| 10. Physical trauma, for example a punch or squeeze. | ☐ | ☐ | ☐ | ☐ | ☐ |
| 11. Using microwave ovens. | ☐ | ☐ | ☐ | ☐ | ☐ |
| 12. Drinking from plastic bottles. | ☐ | ☐ | ☐ | ☐ | ☐ |

This is a picture of 100 people, out of 100 people, how many do you think will develop cancer at some point in their life?

Please write your answer here: ______________


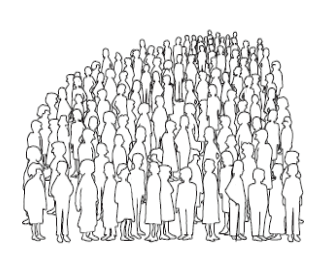


**Section 2 – Attitude and treatment**

Please indicate your agreement with the following statements

When I think of cancer, I automatically think of death.

- Disagree
- Agree

I think cancer can be treated.

- No
- Yes

I know people who have survived cancer.

- No
- Yes

*The following statements are about your attitudes to cancer. Please try to indicate how much you agree or disagree with each of the statements.*

|  | Strongly Disagree | Disagree | Not sure | Agree | Strongly Agree |
| --- | --- | --- | --- | --- | --- |
| 1. Once you’ve had cancer you can never be ‘normal’ again. | ☐ | ☐ | ☐ | ☐ | ☐ |
| 2. Getting cancer means having to mentally prepare oneself for death. | ☐ | ☐ | ☐ | ☐ | ☐ |
| 3. A person with cancer is to blame for their condition. | ☐ | ☐ | ☐ | ☐ | ☐ |
| 4. Having cancer usually ruins a person’s career. | ☐ | ☐ | ☐ | ☐ | ☐ |
| 5. A person with cancer is accountable for their condition. | ☐ | ☐ | ☐ | ☐ | ☐ |
| 6. Cancer usually ruins close personal relationships. | ☐ | ☐ | ☐ | ☐ | ☐ |
| 7. Cancer devastates the lives of those it touches. | ☐ | ☐ | ☐ | ☐ | ☐ |
| 8. A person with cancer is liable for their condition. | ☐ | ☐ | ☐ | ☐ | ☐ |
| 9. If a person has cancer, it’s probably their fault. | ☐ | ☐ | ☐ | ☐ | ☐ |

**Section 3 – Demographics**

How old are you?

__________________

What is your Gender?

- Female
- Male

In which Degree program are you enrolled?

____________________________

Are you a Medical (MBChB) student?

- No
- Yes

Has anyone in your family been diagnosed of Cancer?

- No
- Yes

Are you interested in screening for any cancer?

- No
- Yes
